# SS-OCT-based ocular biometric characteristics of patients with nuclear cataract

**DOI:** 10.1186/s12938-025-01386-5

**Published:** 2025-05-09

**Authors:** Zhao Xu, Ke Yin, Liming Wang, Lijie Dong, Jing Sun, Aihua Liu

**Affiliations:** Tianjin Key Laboratory of Retinal Functions and Diseases, Tianjin Branch of National Clinical Research Center for Ocular Disease, Eye Institute and School of Optometry, Tianjin Medical University Eye Hospital, No. 251 Fukuang Road, Nankai District, Tianjin, China

**Keywords:** Axial length, IOL-Master700, Keratometry, Nuclear cataract, Ocular biometric parameters, Phacoemulsification

## Abstract

**Background:**

This observational, prospective, analytical, and cross-sectional study was designed to systematically analyze ocular biometric parameters in patients with nuclear cataract exhibiting nuclear hardness grade ≥ 3 according to the Emery-Little classification under slit-lamp examination who scheduled for phacoemulsification. Ocular biometric measurements were acquired using the IOL Master 700, a swept-source optical coherence tomography (SS-OCT) device.

**Results:**

Age was negatively correlated with axial length (AL), anterior chamber depth (ACD), white-to-white (WTW), and pupil diameter (PD) (r_AL_ = − 0.13, r_ACD_ = − 0.26, r_WTW_ = − 0.18, all P < 0.001; r_PD_ = − 0.09, P < 0.01) but positively correlated with total steep keratometry (TKs) (r_TKs_ = 0.13, P < 0.001). AL was negatively correlated with total flat keratometry (TKf), TKs, and WTW (r_TKf_ = − 0.3, r_TKs_ = − 0.27, r_WTW_ = − 0.18, all P < 0.001) but positively correlated with ACD and PD (r_ACD_ = 0.43, r_PD_ = 0.10, all P < 0.001). Men had smaller TK and PD but larger WTW than women.

**Conclusions:**

These findings highlight significant sex-related differences in biometric parameters among patients with nuclear cataract, which are closely related to AL and age. Considering variation with these parameters helps personalize surgical plans and prevent complications.

**Supplementary Information:**

The online version contains supplementary material available at 10.1186/s12938-025-01386-5.

## Background

Ocular biometric parameters not only serve to assess the ocular condition of patients, but also guide the selection of intraocular lens (IOL) and predict postoperative complications [1, 2]. However, these biometric parameters are influenced by factors such as environment, region, age, and ethnicity [3–6]. Several studies have indicated that patients with different types of cataracts exhibit varied ocular biometric parameters [7, 8]. Among the most prevalent cataract types worldwide, nuclear cataract is a leading cause of blindness characterized by progressive darkening and hardening of the crystal nucleus area [9]. However, vision improvement and quality of life can be restored through cataract surgery for these patients. Previous studies have shown that inaccuracy in measurement of axial length (AL), keratometry and anterior chamber depth (ACD) can contribute to 36%, 22% and 42% of errors in the calculation of IOL power, respectively [10, 11]. SS-OCT presents non-contact advantages with high precision and good repeatability, being considered the gold standard for biometric measurement [12, 13]. Therefore, the accurate measurement of preoperative ocular biometric parameters and the consideration of their characteristics are crucial for the recovery of optical quality in cataract patients after surgery.

Contrary to current research predominantly focuses on overall analysis and description limited to specific ocular biometric parameters without a focus on specific types of cataracts [3–6, 14–16], the present study independently and relatively comprehensively examined the distribution characteristics and correlations of ocular biometric parameters in patients with nuclear cataracts. The objective of this study was to provide guidance and support for clinical practice based on the gathered data.

## Results

### Population demographics

A total of 1406 eyes were included in this study. Among them, 605 eyes (43.1%) were male and 801 eyes (56.9%) were female. A total of 738 eyes (52.5%) were right eyes, while 668 eyes (47.5%) were left eyes. The mean age of the patients was (70.03 ± 9.96) years. The average age of male patients was (68.83 ± 11.00) years, while female patients was (70.93 ± 9.00) years. Male patients were significantly younger than female patients (P < 0.01).

### Distribution characteristics of ocular biometric parameters

#### Differences in biometric parameters between sexes

The mean AL of 1406 eyes was (23.92 ± 1.85) mm, with males having a significantly longer AL than females (24.20 ± 1.64 vs. 23.71 ± 1.97 mm, P < 0.001). The mean ACD was (3.07 ± 0.47) mm, with males having a deeper ACD (3.20 ± 0.45) mm compared to females (2.97 ± 0.46 mm, P < 0.001). The mean PD was lower in males compared to females (3.53 ± 1.15 vs. 3.69 ± 1.26 mm, P < 0.01), while the mean WTW was higher in males (11.66 ± 0.44) mm than that for females (11.55 ± 0.42 mm, P < 0.001). The mean CCT was (534.25 ± 32.86) μm, with males showing a higher CCT compared to females (538.09 ± 33.40 vs. 531.35 ± 32.17 μm, P < 0.001). The anterior corneal refractive parameters were as follows: the mean AKfm were (43.99 ± 1.63) D, with the AKf and AKs for males at (43.47 ± 1.64) D and (44.56 ± 1.73) D, respectively, which were lower than those for females at (44.37 ± 1.51D, P < 0.001) and AKs at (45.41 ± 1.61D, P < 0.001). The posterior corneal refractive parameters: the mean PKfm was (5.85 ± 0.23) D, with the PKf and PKs for males at (5.78 ± 0.23) D and (6.00 ± 0.25) D, respectively, which were lower than those for females at (5.90 ± 0.22D, P < 0.001) and AKs at (6.15 ± 0.26D, P < 0.001). Total corneal refractive parameters: the mean TKfm was (43.93 ± 1.73) D, with males having lower TKf (43.44 ± 1.70) D and Tks (44.63 ± 1.77) D compared to females (44.30 ± 1.65 D, P < 0.001 for TKf; 45.46 ± 1.76 D, P < 0.001 for TKs), as shown in Table 1. After adjusting for age, significant differences between sexes were still observed for these parameters, except for CCT, as shown in Supplementary Table (S1). Finally, we compared the anterior and total corneal refractive parameters. Our results indicated that AKf and AKs in cataract patients were similar to TKf and TKs. Our findings revealed that the AKf and AKs of cataract patients were similar to the TKf and TKs. However, ACA was found to be smaller than TCA (1.07 ± 0.80 VS. 1.17 ± 0.84D, P < 0.01), as shown in Supplementary Fig (S2).

**Table 1:** Overall and intersex distribution of ocular biometric parameters in patients with nuclear cataract

Ocular parameters	Male	Female	*P* value	Total
Age (yr)	68.83 ± 11.00	70.93 ± 9.00	0.005^b^	70.03 ± 9.96
Axial length (mm)	24.20 ± 1.64	23.71 ± 1.97	0.000^b^	23.92 ± 1.85
Anterior chamber depth (mm)	3.20 ± 0.45	2.97 ± 0.46	0.000^a^	3.07 ± 0.47
Lens thickness (mm)	4.46 ± 0.53	4.51 ± 0.48	0.080^a^	4.49 ± 0.50
White-to-white (mm)	11.81 ± 0.43	11.55 ± 0.42	0.000^b^	11.66 ± 0.44
Central corneal thickness (μm)	538.09 ± 33.40	531.35 ± 32.17	0.000^a^	534.25 ± 32.86
Pupil diameter (mm)	3.53 ± 1.15	3.69 ± 1.26	0.009^b^	3.60 ± 1.21
Angle alpha (mm)	0.46 ± 0.22	0.49 ± 0.28	0.201^b^	0.48 ± 0.25
Angle kappa (mm)	0.30 ± 0.22	0.29 ± 0.21	0.853^b^	0.30 ± 0.21
Mean anterior keratometry (D)	44.00 ± 1.64	44.88 ± 1.50	0.000^a^	44.50 ± 1.62
Anterior flat keratometry (D)	43.47 ± 1.64	44.37 ± 1.51	0.000^a^	43.99 ± 1.63
Anterior steep keratometry (D)	44.56 ± 1.73	45.41 ± 1.61	0.000^a^	45.04 ± 1.72
Anterior corneal astigmatism (D)	1.08 ± 0.78	1.05 ± 0.82	0.294^b^	1.07 ± 0.80
Mean posterior keratometry (D)	5.89 ± 0.23	6.02 ± 0.22	0.000^a^	5.96 ± 0.24
Posterior flat keratometry (D)	5.78 ± 0.23	5.90 ± 0.22	0.000^a^	5.85 ± 0.23
Posterior steep keratometry (D)	6.00 ± 0.25	6.15 ± 0.26	0.000^b^	6.09 ± 0.26
Posterior corneal astigmatism (D)	0.23 ± 0.13	0.25 ± 0.15	0.135^b^	0.24 ± 0.14
Mean total keratometry (D)	44.02 ± 1.69	44.87 ± 1.65	0.000^b^	44.51 ± 1.72
Total flat keratometry (D)	43.44 ± 1.70	44.30 ± 1.65	0.000^b^	43.93 ± 1.73
Total steep keratometry (D)	44.63 ± 1.77	45.46 ± 1.76	0.000^b^	45.10 ± 1.91
Total corneal astigmatism (D)	1.19 ± 0.83	1.16 ± 0.85	0.338^b^	1.17 ± 0.84

^a^ Independent sample t test; ^b^Mann–Whitney U test

#### Ocular biometric parameters with no distribution difference between the sexes

The mean LT was (4.49 ± 0.50) mm, with no significant difference between males and females. The mean Alpha Angle for males was (0.48 ± 0.25) mm, similar to that of females. The mean Kappa Angle was (0.30 ± 0.21) mm, with no significant difference between males and females. The mean CA was (1.07 ± 0.80) D, with no differences between males and females. The mean PCA was (0.24 ± 0.14) D, with similar values for both males and females. Finally, the mean TCA for males was (1.17 ± 0.84) D, with no significant difference from that of females. As shown in Table 1.

### Correlation of ocular biometric parameters

#### Correlation between age and various ocular biometric parameters

Age was negatively correlated with AL (r = − 0.13, P < 0.001), ACD (r = − 0.26, P < 0.001), WTW (r = − 0.18, P < 0.001), and PD (r = − 0.09, P < 0.01). Additionally, a positive correlation was observed with LT (r = 0.34, P < 0.001), angle alpha (r = 0.22, P < 0.001) and angle kappa (r = 0.10, P < 0.001). Further analysis through linear regression revealed that age showed a strong negative linear correlation with AL and ACD, and a positive linear correlation with LT, as shown in Supplementary Fig(S3). Moreover, age was positive correlated with CA and TCA (r = 0.17, r = 022, P < 0.001), as well as Ks and TKs (r = 0.11, r = 0.13, P < 0.001), but no correlation was deserved age and Kf or TKf. No association was found between age and posterior corneal refractive parameters.

#### Correlation between AL and various ocular biometric parameters

AL showed a significant positive correlation with ACD (r = 0.43, P < 0.001), as well as with WTW (r = 0.21, P < 0.001), and with PD (r = 0.10, P < 0.001). It also exhibited a positive correlation with angle alpha (r = 0.21, P < 0.001). Conversely, AL was negatively correlated with LT (r = − 0.11, P < 0.001). Furthermore, AL was negatively correlated with AKf, PKf and TKf (r = − 0.31, r = − 0.30, r = − 0.30, P < 0.001); it was also negatively correlated with AKs, PKs and TKs (r = − 0.27, r = − 0.30, r = − 0.27, P < 0.001). However, there were no correlations between AL and ACA, PCA, or TCA.

#### Correlation analysis of other ocular biometric parameters

**Fig. 1:**
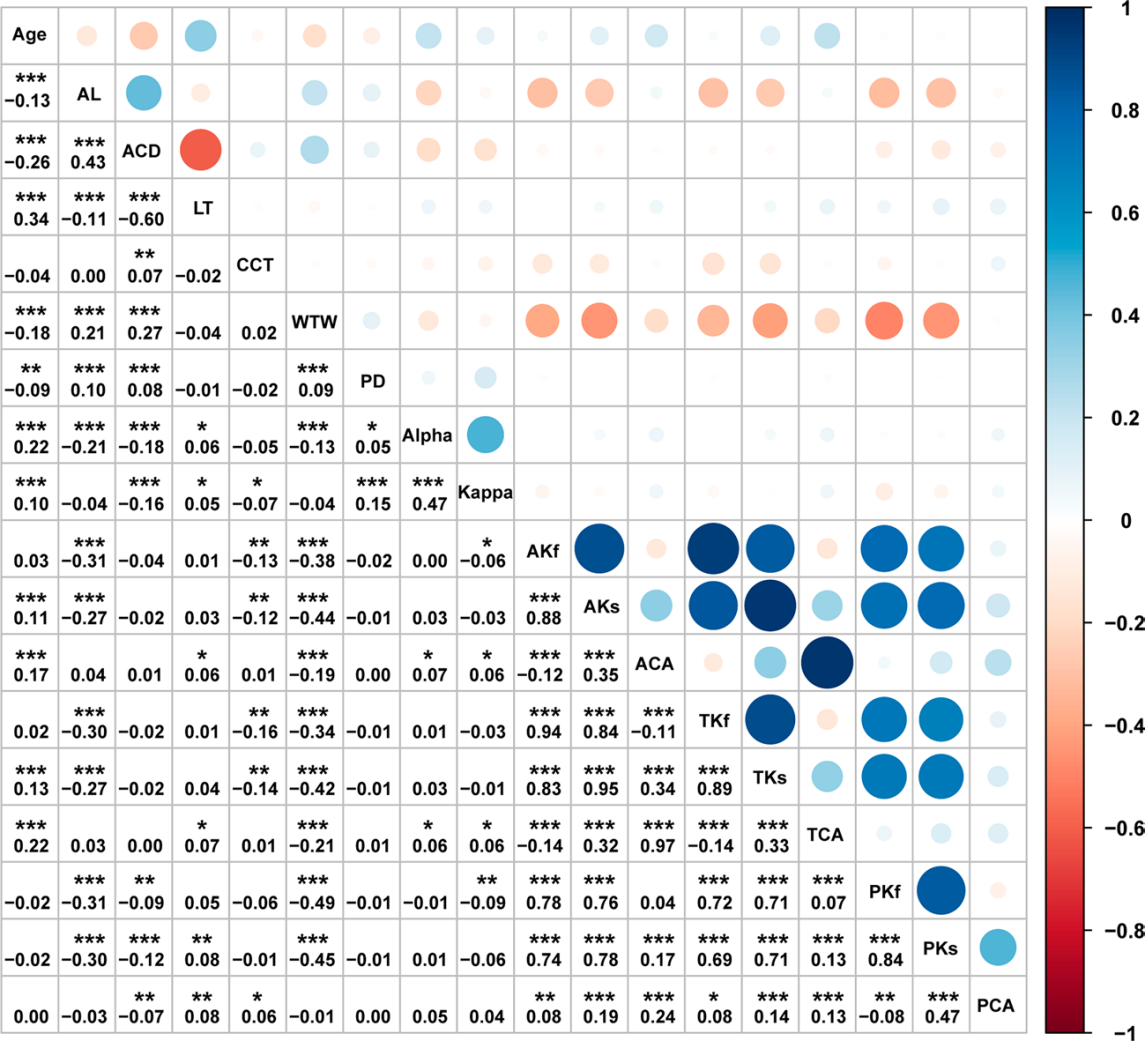
The correlation of various ocular biometric parameters. The color circles in the upper half of the figure represent the values of the correlation coefficients, with strong positive correlations represented by blue and strong negative correlations represented by wine red. The numbers in the lower half of the figure represent correlation, and significance is represented by an asterisk: blank spaces on the numbers indicate no correlation between parameters (P > 0.05), * parameters have correlation (P < 0.05), ** parameters have correlation (P < 0.01), and *** parameters have correlation (P < 0.001)

ACD exhibited a significant negative correlation with LT (r = 0.43, P < 0.001), angle alpha (r = − 0.18, P < 0.001), and angle kappa (r = − 0.16, P < 0.001). ACD also exhibited a negative correlation with PKf, PKs, and PCA (r = − 0.09, r = − 0.12, r = − 0.07, P < 0.01). Conversely, ACD was positively correlated with WTW (r = 0.27, P < 0.001, CCT (r = 0.07, P < 0.01), and PD (r = 0.08, P < 0.00 l). Further analysis confirmed a strong negative linear relationship between ACD and LT, whereas the association between ACD and axial length (AL) appeared to be more complex. Curve fitting analysis revealed that when AL > 25 mm, the rate of ACD change gradually decreases with increasing AL, as illustrated in Supplementary Fig(S2). WTW was positively correlated with PD (r = 0.09, P < 0.001), and angle alpha (r = 0.13, P < 0.001). Furthermore, WTW was found to have no correlation with PCA, but exhibited negative correlations with anterior corneal refractive parameters AKf, AKs, ACA (r = − 0.38, r = − 0.44, r = − 0.19, P < 0.001). Additionally, Additionally, WTW was negatively correlated with posterior corneal refractive parameters PKf and PKs (r = − 0.49, r = − 0.45, r = − 0.07, P < 0.001), and total corneal refractive parameters TKf, TKs and TCA (r = − 0.34, r = − 0.42, r = − 0.21, P < 0.001) (Fig. 1).

### Inter-sex and overall characteristic distribution of ocular biometric parameters across different age groups

**Fig. 2:**
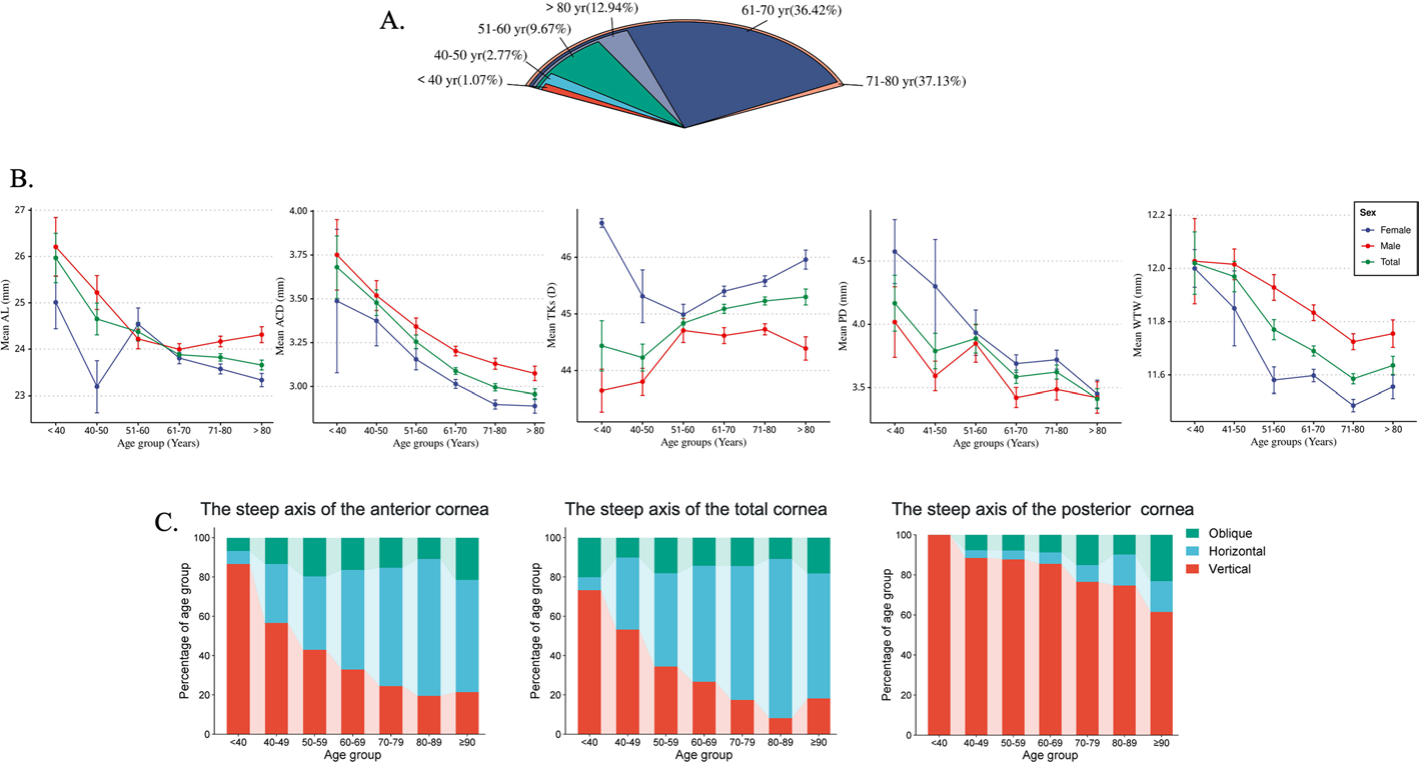
Ocular Biometric Characteristics in Nuclear Cataract Patients by Age. **A** Proportion of patients with nuclear cataract by age group. **B** The distribution of sex-specific biometric parameters within age group. **C** Link fill bar represents the axial distribution of the steep axis of the anterior cornea, total cornea, and posterior cornea for each age group. (Red represents the vertical axis, blue represents the horizontal axis, and green represents the inclined axis)

To further explore the distribution of biometric parameters across different age groups and sexes, patients with nuclear cataract were categorized into age groups: < 40 years old, 40–50 years old, 51–60 years old, 61–70 years old, 71–80 years old, and > 80 years old. The highest proportion was in the 71–80 age group, followed by the 61–79 age group, while the < 40 age group had the lowest proportion (Fig. 2A). Significant Variations in AL, ACD, TKs, PD and WTW distribution were observed across different age groups and sexes. Additionally, the steep axis of the cornea in patients with nuclear cataracts also changes with age (Fig. 2B). In the age-related distribution of the steep axis of the anterior cornea, the vertical axis was predominant in the < 40-year group, followed by the horizontal and oblique axis. With advancing age, the proportion of vertical axis continuously decreases while that of horizontal axis keeps increasing. Although there is a similar trend in steep axis of the total cornea by age as seen in anterior cornea, there are differences in axial distribution within each age group. Regarding the steep axis of the posterior cornea, the axial direction was predominantly horizontal in the < 40-year group. However, with increasing age, there is a gradual decrease in vertical axial proportion and an increase in horizontal axial proportion (Fig. 2C).

### Inter-sex and overall characteristic distribution of ocular biometric parameters across different AL groups

**Fig. 3:**
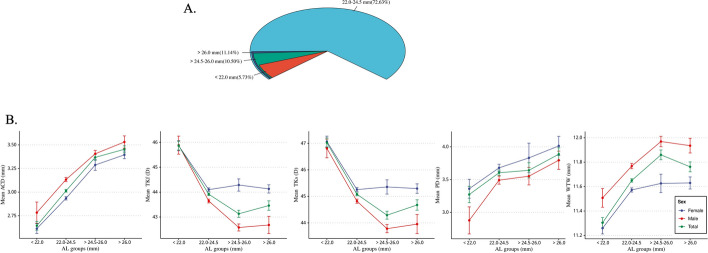
Ocular Biometric Characteristics in Nuclear Cataract Patients by Axial Length (AL) Group. **A** Proportion of patients with nuclear cataract by AL group. **B** The distribution of sex-specific biometric parameters within AL group

AL was categorized into < 22.0 mm, 22.0–24.5 mm, > 24.5–26.0 mm, and > 26.0 mm to investigate the sex-specific distribution of biometric parameters within each AL group. The highest proportion of patients had an AL of 22.0–24.5 mm, followed by those with AL > 26.0 mm, while the proportion of patients with AL > 24.5–26.0 mm was comparable (Fig. 3A). Patients with AL < 22.0 mm comprised the smallest proportion. Significant variations in ACD, TKf, TKs, PD, and WTW distributions were observed across different AL groups and sexes (Fig. 3B).

## Discussion

Accurate calculation of refractive power is a critical determinant of refractive outcomes following phacoemulsification. According to the European Registry of Quality Outcomes for Cataract and Refractive Surgery, 73.7% of eyes achieve a prediction error (PE) within ± 0.5D postoperatively [17], however, this remains below the expectations of patients seeking optimal visual outcomes. Most formulas for IOL power calculation based on ocular biometric parameters are accurate for eyes with AL in the (22.0–25.0) mm range, but their accuracy is doubtful for eyes with longer or shorter AL [18]. Therefore, latest generation of IOL power calculation formulas incorporates additional parameters, including WTW, LT, CCT, to enhance predictive accuracy [19, 20]. Previous studies have indicated that patients with nuclear cataracts typically have longer AL and thicker LT, while patients with posterior subcapsular cataracts usually exhibit shorter AL, deeper ACD, and thinner LT. Patients with cortical cataracts often demonstrate relatively shallow ACD and a smaller ratio of ACD to AL [7]. SS-OCT exhibits superior penetration for cataracts and has a high AL detection rate compared to other biological measurements [21]. Therefore, this study utilized the IOL Master 700 to obtain ocular biometric measurements in patients with nuclear cataracts scheduled for surgery.

As a critical ocular biometric parameter, AL is fundamental in diagnosing pathological axial myopia, selecting IOL calculation formulas, and predicting postoperative IOL displacement [22, 23]. AL of male patients with nuclear cataracts is longer than that of females. Some studies suggest that this difference may be attributed to height variation [24]. However, a study found that even after adjusting for height through multivariable analysis, significant sex-related differences in AL persisted, indicating that sex may be an independent determinant of AL [25]. Preoperative AL can affect the tilt and decentration of IOL after implantation. Gu et al. [23] reported that patients with shorter or longer AL tend to exhibit greater tilt and decentration of the IOL postoperatively. In our study, it was found that 11.14% of cataract patients had AL ≥ 26 mm, while 5.73% had AL ≤ 22 mm. In our study, 11.14% of cataract patients had AL ≥ 26 mm, while 5.73% had AL ≤ 22 mm. Given the strong correlation between AL, age, and sex, these factors should be carefully considered when selecting IOL power to minimize the risk of postoperative displacement.

Corneal curvature plays a crucial role in eye refraction. Unlike conventional keratometry calculations, IOL Master 700 can measure both the axial direction of keratometry and total keratometry by incorporating anterior and posterior keratometry along with CCT [26]. Firstly, we analyzed the distributional differences between anterior and total corneal refractive parameters. The results demonstrated that AKf and TKf, as well as AKs and TKs, followed similar trends. Furthermore, there was a strong positive correlation between ACA and TCA. Both ACA and TCA exhibited an increasing trend with age. These results align with previous research demonstrating that total corneal curvature shifts are predominantly driven by morphological alterations in the anterior corneal surface [26, 27]. However, a 0.1D difference between ACA and TCA suggest that the posterior corneal refractive status plays a certain role in TCA. Notably, all refractive parameters except PKf showed significant correlations with TCA, implying that PKf may be a key contributor to the ACA-TCA discrepancy. In our study, 4% of patients exhibited PCA > 0.5D, while 47% had TCA > 1.0D, emphasizing the need for astigmatism correction and the critical role of posterior corneal astigmatism assessment. Additionally, males had significantly lower AKf/Pkf and AKs/PKs values than females, whereas CA showed no sex-related differences. Correlation analysis revealed that as AL increased, Ks and Kf progressively flattened, while CA remained unassociated associated with AL. This phenomenon may be attributed to ocular anatomical and physiological mechanisms, where reduced corneal curvature with increasing AL helps maintain visual function [28]. However, the AL-corneal curvature relationship alone does not fully explain sex differences in Ks and Kf, suggesting the importance of incorporating sex as a variable in IOL power calculation formulas to enhance predictive models. Furthermore, with aging, AKs increase alongside ACA, whereas AKf remained stable. Thus, age-related changes in Aks may primarily drive ACA increases. Additionally, with aging, the steep axis of astigmatism tended to shift progressively from vertical to horizontal. However, no significant correlation was observed between age and posterior corneal refractive parameters, indicating that posterior corneal refractive status remains stable over time. Understanding age-related astigmatism changes offers critical insights for personalized surgical incision planning and optimized astigmatism correction. In summary, our findings underscore the importance of integrating age- and sex-related astigmatism changes into surgical planning.

WTW is a key parameter in diagnosing congenital glaucoma, micro- and megalocornea. It has also gained significance in refractive cataract surgery [29–31]. In nuclear cataracts patients, greater WTW is associated with male sex, younger age, longer AL, deeper ACD, and flatter corneas. Contrary to previous research regarding the correlation between WTW and LT, ACD [30], our study found no correlation between LT and CCT with WTW. This discrepancy may stem from differences in study populations. Previous studies have indicated that LT is significantly greater in nuclear cataracts than in posterior subcapsular cataracts. However, our study focused exclusively on nuclear cataracts rather than all cataract types [7]. Additionally, patients with smaller Alpha angles exhibited larger WTW. Thus, future studies on WTW associations should account for cataract subtype variations.

PD, Angle Alpha, and Angle Kappa are key preoperative parameters used to guide the implantation of multifocal intraocular lenses (MIOL) in cataract patients [1]. Patients with nuclear cataracts who are female, younger and have longer AL tend to have larger PD. Literature reports that over 42% of patients with larger PD may develop severe glare and reduced high-frequency contrast sensitivity postoperatively [32]. Therefore, greater attention should be given to optimizing visual quality in these patients. Notably, both Angle Kappa and Angle Alpha are independent of sex and exhibit a significant positive correlation. However, unlike Angle Alpha, Angle Kappa is not related to AL and WTW. Other studies have reported that cataract patients with a preoperative Angle Kappa > 0.4 mm are at increased risk of severe glare following MIOL implantation [33]. The current consensus in China on MIOL implantation recommends an Angle Kappa < 0.5 mm or less than half the diameter of the central refractive optical zone in MIOL [1]. Additionally, a large Angle Alpha may lead to postoperative IOL misalignment. Therefore, when selecting Toric IOL or MIOL, careful assessment of whether Angle Alpha exceeds 0.5 mm is necessary [2].

The current study has some limitations, primarily due to its hospital-based design, which does not fully represent the entire regional cataract patient population. Additionally, this study exclusively included patients with nuclear cataracts who underwent phacoemulsification. All included cases had a nuclear hardness grade exceeding 3. However, statistical methods were not performed to determine the mean and standard deviation of nuclear hardness grades. As a result, a comprehensive analysis of the correlations between nuclear cataract grading and ocular biometric parameters could not be performed. Lastly, the cross-sectional design of this study limits the interpretation of correlations, as they only reflect aging-related trends in older patients and do not provide insights into longitudinal changes in younger individuals. In response to these issues, we will conduct a larger-scale, community-based epidemiologic survey for further investigation.

## Conclusions

In conclusion, this study systematically analyzed the ocular biometric characteristics of nuclear cataracts. The findings may assist clinicians in optimizing surgical plans and minimizing complications. Additionally, we identified significant sex-based differences in ocular parameters and established novel correlations between ocular biometric parameters, key demographic factors (age/sex) and AL, providing a stratified reference for personalized formula selection. Integrating anatomical parameters such as WTW, PD and TKs alongside demographic variables, our nuclear cataract-specific analysis may improve the precision of IOL power calculations.

## Methods

### Study population

This study included patients with nuclear cataract admitted to Tianjin Medical University Eye Hospital between January 2023 and December 2023 who were scheduled for cataract surgery. All participants were evaluated for lens opacity using the Lens Opacities Classification System III, and nuclear density was graded according to the Emery-Little classification under slit-lamp examination. The inclusion criteria comprised patients diagnosed with nuclear cataract presenting a nuclear hardness grade of ≥ 3, while exclusion criteria included a history of ocular surgery, fundus disease, and corneal conditions including keratitis, keratoconus, corneal dystrophy, and severe untreated dry eye. Additionally, patients who had worn soft contact lenses within 1 week or hard contact lenses within 1 month were excluded. These exclusion criteria were intended to ensure accurate acquisition of ocular biometric parameters. This study was approved by the Ethics Committee of Tianjin Medical University Eye Hospital. All procedures were performed in accordance with the Declaration of Helsinki [2021KY(L)-33]. Written informed consent was waived for this study in accordance with national legislation and institutional requirements.

### Examination procedures

Ocular biometric parameters were measured with the IOL Master 700 biometry system (Carl Zeiss, Germany), a device employing SS-OCT technology. The measured parameters include AL (the distance from the tear film surface to the retinal pigment epithelium in the macular region along the optic axis), ACD (the distance from the corneal front surface to the anterior lens surface cornea), lens thickness (LT), corneal limbus white to white distance (WTW), central corneal thickness (CCT), the pupil diameter (PD, measured under natural light); Alpha Angle (the eccentricity of the optic axis center relative to the cornea center) and Kappa Angle (the eccentricity of the optic axis center relative to the pupil center); And various corneal curvature parameters: anterior steep keratometry (AKs), anterior flat keratometry (AKf), mean anterior keratometry (AKm), anterior corneal astigmatism (ACA); posterior steep keratometry (PKs), posterior flat keratometry (PKf), mean posterior K (PKm), posterior corneal astigmatism (PCA); total steep keratometry (TKs), total flat keratometry (TKf), mean total keratometry (TKm), total corneal astigmatism (TCA), and steep axis direction. Patients were divided into Vertical (60° to 120°), oblique (31° to 59° or 121° to 149°) and Horizontal (0° to 30° or 150° to 180°) according to the maximum corneal refractive power diameters. All examinations were conducted by a single experienced technician who received standardized training on the measurement protocol prior to study commencement. Three consecutive measurements were obtained for each parameter, with the average value used for analysis to minimize intra-observer variability.

### Statistical analysis

Statistical analysis was performed using SPSS (Version 26, IBM Corporation). The Kolmogorov–Smirnov test (K-S test) was employed to assess data normality, while sex differences were analyzed using the Chi-square (*χ*^*2*^) test. Differences in ACD, LT, CCT, AKm, AKs, PKm, and PKf between sexes were measured using an independent sample t-test, while differences in Age, AL, WTW, PD, Angle alpha, Angle kappa, ACA, PKs, PCA, TKm, TKf, TCA were measured using the Mann–Whitney U test. Analysis of covariance (ANCOVA) was performed to determine whether significant sex differences in parameter distributions persisted after adjusting for age as a covariate. Spearman correlation analysis was conducted to evaluate the associations among ocular biometric parameters, with r denoting the correlation coefficient. Statistical significance was defined as P < 0.05.

## Supplementary Information

Supplementary material 1. Supplementary Tab(S1). The distribution of ocular biometric parameters between sexes after adjusting for age.

Supplementary material 2. Supplementary Fig(S2). The comparison of the anterior corneal refractive parameters and total corneal refractive parameters.

Supplementary material 3 .Supplementary Fig(S3). Linear Regression Analysis of Ocular Biometric Parameters in Nuclear Cataract Patients. (A),( B), and (C) represent the linear regression scatter plot of age with axial Length, anterior chamber depth, and lens thickness. (C), (D) represent Linear regression scatter plot of anterior chamber depth with axial length and lens thickness (red represents linear regression fitting line, black represents LOESS fitting curve).

## Data Availability

The data used to support the findings of this study are available from the corresponding author upon reasonable request.

## Abbreviations

AL: Axial length
ACD: Anterior chamber depth
WTW: White-to-white
PD: Pupil diameter
TKs: Total steep keratometry
TKf: Total flat keratometry
IOL: Intraocular Lens
SS-OCT: Swept-source optical coherence tomography
LT: Lens thickness
CCT: Central corneal thickness
AKs: Anterior steep keratometry
AKf: Anterior flat keratometry
AKm: Mean anterior keratometry
ACA: Anterior corneal astigmatism
PKs: Posterior steep keratometry
PKf: Posterior flat keratometry
PKm: Mean posterior K
PCA: Posterior corneal astigmatism
TKm: Mean total keratometry
TCA: Total corneal astigmatism
