# Supplementary material for: SS-OCT-based ocular biometric characteristics of patients with nuclear cataract
Source: Biomed Eng Online. 2025 May 9;24:56. doi: 10.1186/s12938-025-01386-5 (PMC12065238; doi:10.1186/s12938-025-01386-5)
Supplement: Supplementary file 1 — Supplementary material 1. Supplementary Tab(S1). The distribution of ocular biometric parameters between sexes after adjusting for age. [file 12938_2025_1386_MOESM1_ESM.docx]

**Supplementary Tab(S1)** The distribution of ocular biometric parameters between sexes after adjusting for age f

| Ocular parameters | Male | Female | F | *P* value |
| --- | --- | --- | --- | --- |
| Axial length（mm） | 24.20±1.64 | 23.71±1.97 | 79.31 | ＜0.001 |
| Anterior chamber depth（mm) | 3.20±0.45 | 2.97±0.46 | 41.46 | ＜0.001 |
| White-to-white (mm) | 11.81±0.43 | 11.55±0.42 | 45.26 | ＜0.01 |
| Central corneal thickness (μm) | 538.09±33.40 | 531.35±32.17 | 3.75 | 0.053 |
| Pupil diameter（mm） | 3.53±1.15 | 3.69±1.26 | 5.99 | 0.015 |
| Anterior flat keratometry（D） | 43.47±1.64 | 44.37±1.51 | 30.28 | ＜0.001 |
| Anterior steep keratometry（D） | 44.56±1.73 | 45.41±1.61 | 20.61 | ＜0.001 |
| Posterior flat keratometry（D） | 5.78±0.23 | 5.90±0.22 | 23.93 | ＜0.001 |
| Posterior steep keratometry（D） | 6.00±0.25 | 6.15±0.26 | 28.61 | ＜0.001 |
| Total flat keratometry（D） | 43.44±1.70 | 44.30±1.65 | 18.90 | ＜0.001 |
| Total steep keratometry（D） | 44.63±1.77 | 45.46±1.76 | 12.53 | ＜0.001 |
