# Supplementary figures and images for: SS-OCT-based ocular biometric characteristics of patients with nuclear cataract
Source: Biomed Eng Online. 2025 May 9;24:56. doi: 10.1186/s12938-025-01386-5 (PMC12065238; doi:10.1186/s12938-025-01386-5)

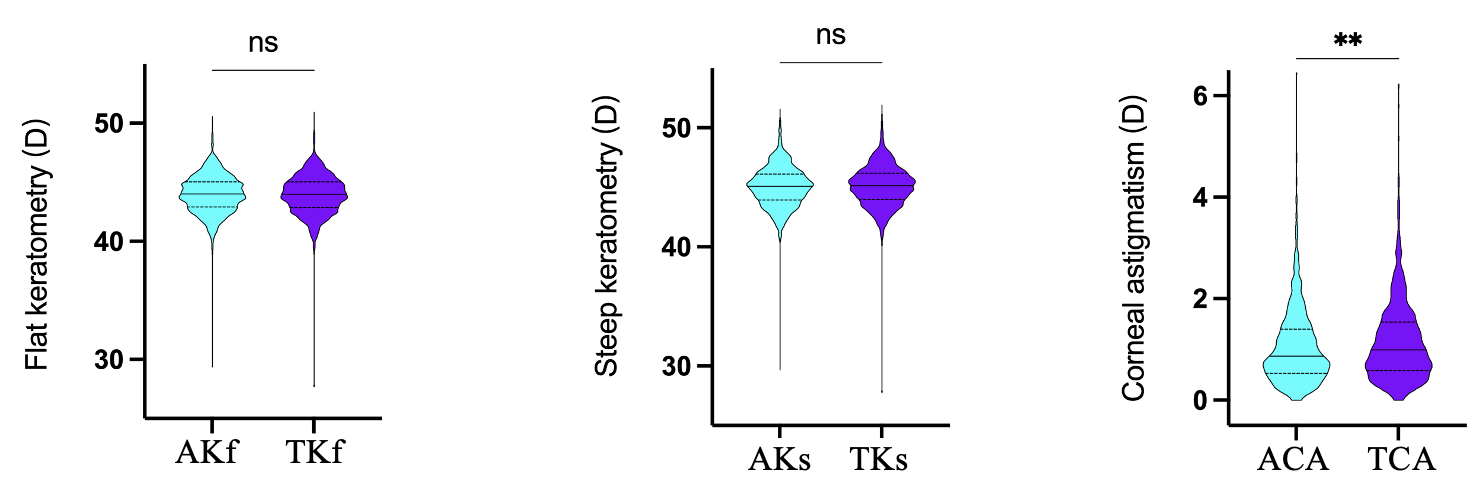

Supplement: Supplementary file 2 — Supplementary material 2. Supplementary Fig(S2). The comparison of the anterior corneal refractive parameters and total corneal refractive parameters. [file 12938_2025_1386_MOESM2_ESM.tif]

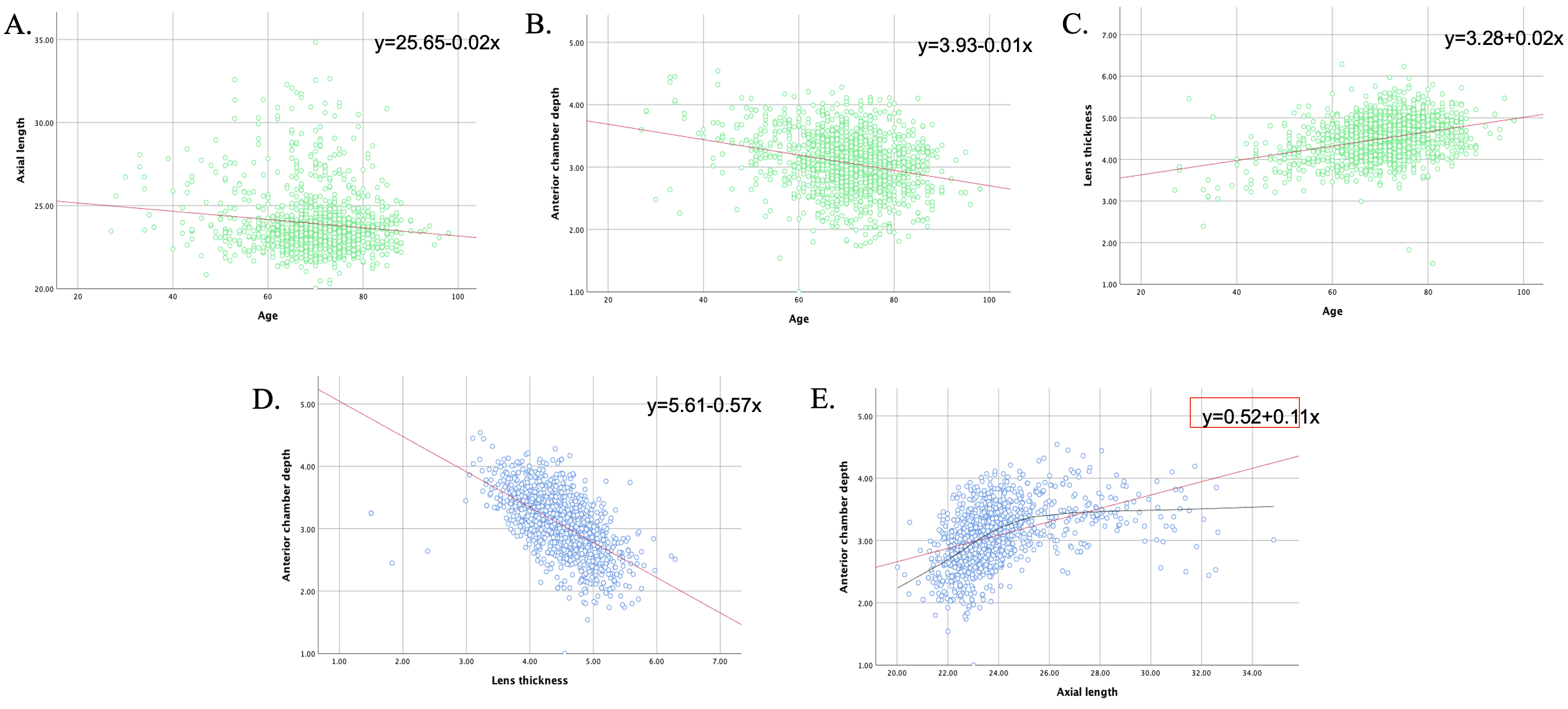

Supplement: Supplementary file 3 — Supplementary material 3 .Supplementary Fig(S3). Linear Regression Analysis of Ocular Biometric Parameters in Nuclear Cataract Patients. (A),( B), and (C) represent the linear regression scatter plot of age with axial Length, anterior chamber depth, and lens thickness. (C), (D) represent Linear regression scatter plot of anterior chamber depth with axial length and lens thickness (red represents linear regression fitting line, black represents LOESS fitting curve). [file 12938_2025_1386_MOESM3_ESM.tif]
